# Isolation, Characterization, and Antimicrobial Activity of Bacterial and Fungal Representatives Associated With Particulate Matter During Haze and Non-haze Days

**DOI:** 10.3389/fmicb.2021.793037

**Published:** 2022-01-11

**Authors:** Dong Yan, Tao Zhang, Jing-Lin Bai, Jing Su, Li-Li Zhao, Hao Wang, Xiao-Mei Fang, Yu-Qin Zhang, Hong-Yu Liu, Li-Yan Yu

**Affiliations:** ^1^China Pharmaceutical Culture Collection, Institute of Medicinal Biotechnology, Chinese Academy of Medical Sciences and Peking Union Medical College, Beijing, China; ^2^Xinxiang Key Laboratory of Pathogenic Biology, Department of Pathogenic Biology, School of Basic Medical Sciences, Xinxiang Medical University, Xinxiang, China

**Keywords:** airborne bacteria and fungi, particulate matter, haze, isolate collection, antimicrobial activity

## Abstract

Particulate matter (PM) has been a threat to the environment and public health in the metropolises of developing industrial countries such as Beijing. The microorganisms associated with PM have an impact on human health if they are exposed to the respiratory tract persistently. There are few reports on the microbial resources collected from PM and their antimicrobial activities. In this study, we greatly expanded the diversity of available commensal organisms by collecting 1,258 bacterial and 456 fungal isolates from 63 PM samples. A total of 77 bacterial genera and 35 fungal genera were included in our pure cultures, with *Bacillus* as the most prevalent cultured bacterial genus, *Aspergillus*, and *Penicillium* as the most prevalent fungal ones. During heavy-haze days, the numbers of colony-forming units (CFUs) and isolates of bacteria and fungi were decreased. *Bacillus, Paenibacillus*, and *Chaetomium* were found to be enriched during haze days, while *Kocuria*, *Microbacterium*, and *Penicillium* were found to be enriched during non-haze days. Antimicrobial activity against common pathogens have been found in 40 bacterial representatives and 1 fungal representative. The collection of airborne strains will provide a basis to greatly increase our understanding of the relationship between bacteria and fungi associated with PM and human health.

## Introduction

In recent years, frequent haze caused mainly by particulate matter (PM) has been a severe problem threatening public health in north China (Cheng et al., 2013; Cao et al., 2014; Gao et al., 2015; Tan et al., 2016; Yan et al., 2016, 2018; Guo et al., 2020; Yang et al., 2021; Zhao et al., 2021). PM is divided into three categories: total suspended particulates (TSP), PM10 (particulate size smaller than 10 μm), and PM2.5 (particulate size smaller than 2.5 μm), which are a mixture of inorganic, organic, and biological components (Van Dingenen et al., 2004). Various PM fractions show significantly different compositions (Zhang et al., 2006). Previous studies have indicated that PM has an important role in air pollution, visibility reduction, and climate change (Booth et al., 2012; Wang et al., 2012; Randles et al., 2013; Shimadera et al., 2013). More importantly, PM has been shown to increase the morbidity and mortality from stroke (Huang et al., 2019), respiratory disease (Zhang and Cao, 2015), heart disease (Parker et al., 2018; Li et al., 2020a), and lung cancer (Hamra et al., 2014; Li et al., 2020b).

Microorganisms associated with PM are known as bioaerosols in the atmosphere, which contribute up to 25% of aerosolized matter (Jaenicke, 2005). Airborne microorganisms may play an important role in human health, either as human pathogens or allergens (Vermani et al., 2010; Yamamoto et al., 2012; Haas et al., 2013). More microbial pathogens and allergens have been linked to higher levels of PM pollution (Cao et al., 2014). The diversity and community composition of PMs-associated airborne microorganisms have been revealed by high-throughput sequencing in China (Woo et al., 2013; Cao et al., 2014; Wei et al., 2016; Yan et al., 2016, 2018; Zhong et al., 2019; Yu et al., 2020), United States (Bowers et al., 2011a,b, 2013; Be et al., 2015), Italy (Franzetti et al., 2011; Bertolini et al., 2013; Romano et al., 2020), Antarctica (Bottos et al., 2014), and Spain (Barberan et al., 2014). Despite the fact that the culture-independent method can reflect the diversity of airborne microbial community rapidly, the roles and functions of culturable microorganisms which can show the potential ability in human health and atmospheric chemistry are neglected. Using culture-dependent methods, the concentrations and composition of the culturable microbial community in the atmosphere have been investigated (Haas et al., 2013; Alghamdi et al., 2014; Fang, 2014; Li et al., 2015; Niazi et al., 2015; Oh et al., 2015; Dong et al., 2016; Okubo et al., 2017; Yuan et al., 2017). However, the microbial communities in various PM fractions (TSP, PM10, and PM2.5) have rarely been combined for study, and thus are not well understood during haze and non-haze days. We can obtain the microbial strains for further study of the metabolic functions of airborne microorganisms using the culture-dependent method.

There is a continuous challenge for novel antibiotics to overcome the serious problem of evolving pathogens, naturally resistant bacteria and fungi, and multidrug resistance among common microbial pathogens (Alanis, 2005; Sharma et al., 2011; Shah et al., 2017). This crisis has prompted experts to call for the revival of natural product drug discovery (Lewis, 2016). However, the enormous known compounds in the background covered the new ones and presents a serious barrier to discovery (Lewis, 2016). Despite this, recent results like those presented by Maffioli et al. (2017) bring hope to the search for new leads from microbial-extract screening. They reported that a nucleoside-analog inhibitor, pseudouridimycin, inhibited bacterial RNA polymerase and exhibits antibacterial activity against drug-resistant bacterial pathogens (Maffioli et al., 2017). The novel microorganisms with unique metabolic properties may be selected by the extreme conditions with wide-ranging and fluctuating temperatures, high levels of solar irradiation, strong oxidizing, and dryness in the atmosphere (Polymenakou, 2012). Nonetheless, there is a scarce number of studies involving microorganisms collected from the air in antimicrobial activity screening (Sobral et al., 2017).

Microbial resources in the atmosphere are a great treasure, while there are few studies on the isolation of airborne microorganisms, especially microorganisms associated with PM. As we know, no study reported that whether the haze influenced the culturable microorganisms in three types of PM fractions, and anti-microbial activities of microorganisms were screened from the PM. The aim in this study is to address the following questions: (1) what are the concentrations of culturable microbial colonies in various haze levels? (2) Does the culturable microbial community composition differ among various PM samples, or different haze-level samples? (3) Which isolated strains from PM samples show anti-microbial activities against common pathogens?

## Materials and Methods

### Sample Collection

PM2.5 samples (the samples containing particulates smaller than 2.5 μm), PM10 samples (the samples containing particulates smaller than 10 μm), and TSP samples (the samples containing total suspended particulates) were collected from the roof of the Conference Building at the Institute of Medicinal Biotechnology, Chinese Academy of Medical Sciences (39°52′43″N, 116°23′21″E, ∼8 m above the ground, ∼400 m from Temple of Heaven Park), an area without major industrial pollution sources nearby. We collected 63 PM samples for 21 days (September 2014 to November 2014) during various haze levels. Sampling was conducted by three portable ambient air samplers (AirMetrics, United States): the impactors were removed from the filter holder of the first for TSP samples, the second was assembled with PM10 impactor for PM10 samples, and the third was assembled with both PM10 and PM2.5 impactors for PM2.5 samples. Ambient air was drawn at an average flow rate of 5 L/min for 24 h per sampling day. PM samples were collected on 47-mm quartz aerosol collection filters (Pall, United States). All the filters were sterilized by autoclaving at 121°C for 20 min before sampling. The filter holders were cleaned with 75% ethanol and all the tools used for changing new filters were autoclaved to avoid contamination (Yan et al., 2016). After sampling, the filters were cut into small pieces and kept in a 2-mL centrifuge tube with 1 mL of saline.

The air quality index (AQI) which is an index for reporting air quality (Yang et al., 2016) was used to indicate the haze level. We defined a day with AQI lower than 100 as a non-haze day, that with AQI in the range of 100–200 as a light-haze day, that with AQI higher than 200 as a heavy-haze day. The environmental parameters such as CO, SO_2_, and NO_2_ were recorded from the monitoring data of Temple of Heaven Park site (∼800 m from sampling site) of Beijing Municipal Environmental Monitoring Center^1^. Temperature (Temp) and relative humidity (RH) were recorded according to the reports of the Chinese National Meteorological Center^2^. The environmental parameters are listed in Supplementary Table 1.

### Isolation and Cultivation of Airborne Bacteria and Fungi

After sampling, the samples were transferred to the laboratory as soon as possible and vortexed for 10 min at first to make the bioaerosol evenly distribute in the saline. Due to the low concentration of airborne microorganisms, 1 mL of PM2.5 samples were diluted with 500 μL of saline, and 500 μL of PM10 samples and 500 μL of TSP samples were diluted with 1 mL of saline, respectively. Then 100 μL of each diluted PM suspension were spread-plated onto four kinds of medium plates, namely; nutrient agar medium (NA) (Haibo, China), tryptic soy agar medium (TSA) (Haibo, China) for the cultivation of bacteria, sabouraud dextrose agar medium (SDA) (OXOID, United Kingdom), and potato dextrose agar medium (PDA) (OXOID, United Kingdom) for cultivation of fungi. Three replicates of each PM sample were spread-plated in each medium. The medium NA and TSA were added with nystatin (0.05 mg/mL) to inhibit the growth of fungi, while SDA and PDA were added with tetracycline (0.05 mg/mL) and streptomycin sulfate (0.05 mg/mL) to inhibit the growth of bacteria. Plates for the cultivation of bacteria were then incubated at 37°C and plates for the cultivation of fungi were incubated at 28°C. After 5 days of incubation, the number of colony-forming units (CFUs) were counted. From each plate, all phenotypically distinct colonies were picked onto fresh media for isolation. Single colonies were picked and re-streaked at least three times to isolate individual strains. Strains were grown in the corresponding medium on the agar slant and temperature conditions and frozen at –80°C in 20% glycerol. A subset of representative isolates (Supplementary Table 2) has been deposited at China Pharmaceutical Culture Collection and the CAMS Collection Center of Pathogenic Microorganisms, Division for Medicinal Microorganisms Related Strains.

### Identification of Airborne Bacteria and Fungi

Genomic DNA of the strains on the agar slant was extracted using Chelex-100 method. The bacterial 16S rRNA gene was amplified by PCR (95°C for 5 min, followed by 30 cycles at 95°C for 30 s, 59°C for 60 s, and 72°C for 90 s and a final extension at 72°C for 10 min) using primers 27F (5′-AGAGTTTGATCCTGGCTCAG-3′) and 1492R (5′-TACGGCTACCTTGTTACGACTT-3′) (Kanagawa, 2003). The fungal internal transcribed spacer (ITS) regions were amplified by PCR (95°C for 5 min, followed by 35 cycles at 95°C for 30 s, 55°C for 45 s, and 72°C for 45 s and a final extension at 72°C for 10 min) using primers ITS1 (5′-TCCGTAGGTGAACCTGCGG-3′) and ITS4 (5′- TCCTCCGCTTATTGATATGC-3′) (White et al., 1989). PCR products were purified and Sanger sequenced at Sangon Biotech (Sangon, Shanghai, China). The 60 bp of the beginning 16S rRNA reads were cut off and cut into 660 bp to ensure the accuracy of the sequences. Similarly, the 60 bp of the beginning ITS reads were cut off and cut into 410 bp with Bioedit software. Thus the bacterial sequences were compared with available 16S rRNA gene sequences from GenBank using the BLAST program and a web-based tool at EzTaxon^3^ as described by Yoon et al. (2017) to determine the approximate phylogenetic affiliation. The top hit from BLAST analysis and GenBank was used to determine fungal species.

### Processing and Statistical Analyses of Sequencing Data

Isolates were clustered to operational taxonomic units (OTUs) with 100% similarity cutoff. The OTUs were aligned using the default settings of MAFFT online (Katoh et al., 2017). The evolutionary trees were constructed using MEGA software version 7 on the basis of the neighbor-joining method (Sudhir et al., 2016) and visualized using Interactive Tree Of Life (iTOL^4^) (Ivica and Peer, 2019). The canonical correspondence analysis (CCA) and permutation test were conducted using R software (version 3.7.0) with the vegan package (R Core Team, 2018). Bubble plots, bar plots, line plots, and violin plots were created using ggplot2 package in the R software. *Post hoc* tests for ANOVA were performed using Statistical Analysis of Metagenomics Profiles software (STAMP) (Parks et al., 2014) to identify significantly different genera among non-, light, and heavy-haze days.

### Screening for Antimicrobial Activity

The antimicrobial activities of the isolates were tested against four type strains of common pathogen, *Escherichia coli* ATCC 25922, *Enterococcus faecalis* ATCC 29212, *Klebsiella pneumonia* ATCC 700603, *Candida albicans* ATCC 10231, using the Kirby–Bauer method (Patel, 2014). The bacterial representatives (except Actinobacteria) were cultivated in B1 and B2 medium while the representatives of Actinobacteria in A1 and A2 medium at 28°C with shaking at 200 rpm for 72 h. The fungal representatives were cultivated in F1 and F2 medium (Supplementary Table 3) at 28°C with shaking at 200 rpm for 96 h. The fermentation broths were centrifuged at 12,000 rpm for 10 min and the supernatant was transferred to a 1.5 mL sterilized tube carefully without disturbing the bacterial pellets. To prepare test plates of the type strains, *E. faecalis* ATCC 29212 were cultured onto brain-heart infusion agar (Haibo, China) plates, *E. coli* ATCC 25922 and *K. pneumonia* ATCC 700603 were cultured onto Mueller–Hinton agar (AOBOX, China) plates for 18–24 h at 37°C. *Candida albicans* ATCC 10231 were cultured onto modified Thayer-Martin agar (Haibo, China) plates for 18–24 h at 28°C. Pure colonies from these plates were cultured in corresponding broth for 4–6 h, adjusted to a 0.5 McFarland turbidity standard suspension which was diluted to 1,000 times in the agar medium, and then poured into petri plates. Each sterile disc was impregnated with 40 μL of the fermentation supernatant, placed, and incubated on the corresponding agar at 37°C for 16–24 h (except for *Candida albicans*, which was cultured at 28°C for 16–24 h) and evaluated for inhibition zones.

## Results

### The Effects of Haze on the Concentration of Culturable Bacteria and Fungi Associated With Particulate Matter

Quartz aerosol collection filters were arranged from Level 1–6 in Supplementary Figure 1A, which showed more PMs were collected as the increasing of haze level. 63 PM samples (21 samples in PM2.5, PM10, and TSP samples, respectively) were collected during haze and non-haze days, including 9 samples in Level 1-days, 12 samples in Level 2-days, 9 samples in Level 3-days, 9 samples in Level 4-days, 9 samples in Level 5-days, 15 samples in Level 6-days (Table 1 and Supplementary Figure 1B).

**TABLE 1:** Sample information in haze and non-haze days.

Sample ID	Sample date	PM2.5 (μg/m^3^)	PM10 (μg/m^3^)	AQI	Haze level	Haze severity
1-PM2.5	9/16/2014	43	81	66	2	Non
1-PM10						
1-TSP						
2-PM2.5	9/17/2014	60	122	87	2	Non
2-PM10						
2-TSP						
3-PM2.5	9/18/2014	78	121	104	3	Light
3-PM10						
3-TSP						
4-PM2.5	9/20/2014	131	204	173	4	Light
4-PM10						
4-TSP						
5-PM2.5	9/21/2014	87	140	116	3	Light
5-PM10						
5-TSP						
6-PM2.5	9/28/2014	99	98	74	2	Non
6-PM10						
6-TSP						
7-PM2.5	9/29/2014	20	43	43	1	Non
7-PM10						
7-TSP						
8-PM2.5	9/30/2014	53	96	74	2	Non
8-PM10						
8-TSP						
9-PM2.5	10/8/2014	278	348	328	6	Heavy
9-PM10						
9-TSP						
10-PM2.5	10/9/2014	301	331	352	6	Heavy
10-PM10						
10-TSP						
11-PM2.5	10/12/2014	8	182	15	1	Non
11-PM10						
11-TSP						
12-PM2.5	10/17/2014	115	176	151	4	Light
12-PM10						
12-TSP						
13-PM2.5	10/18/2014	253	325	303	6	Heavy
13-PM10						
13-TSP						
14-PM2.5	10/19/2014	160	218	211	5	Heavy
14-PM10						
14-TSP						
15-PM2.5	10/20/2014	110	107	144	3	Light
15-PM10						
15-TSP						
16-PM2.5	10/23/2014	153	199	204	5	Heavy
16-PM10						
16-TSP						
17-PM2.5	10/30/2014	162	149	213	5	Heavy
17-PM10						
17-TSP						
18-PM2.5	10/31/2014	132	142	175	4	Light
18-PM10						
18-TSP						
19-PM2.5	11/1/2014	10	30	31	1	Non
19-PM10						
19-TSP						
20-PM2.5	11/19/2014	266	322	316	6	Heavy
20-PM10						
20-TSP						
21-PM2.5	11/20/2014	324	383	374	6	Heavy
21-PM10						
21-TSP						

**FIGURE 1:**
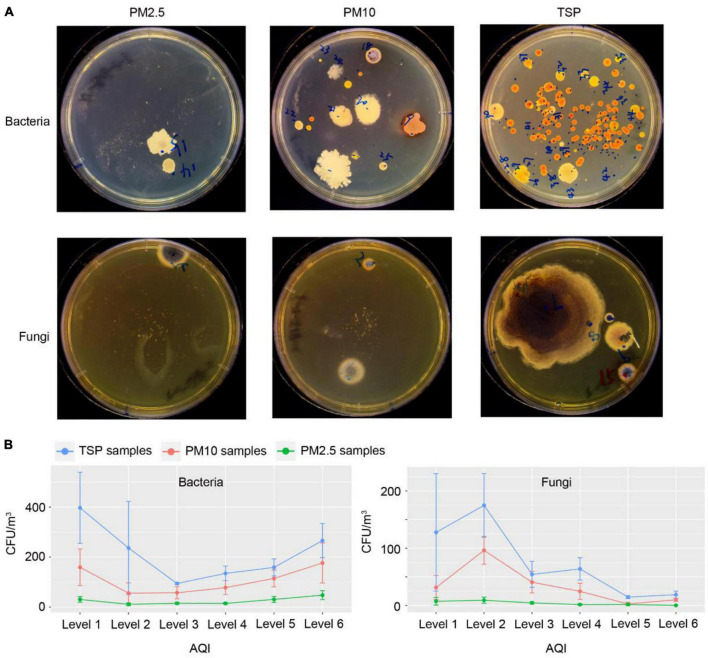
Summary of PM samples and isolation numbers of microbial colonies during various haze-level days. **(A)** Example nutrient agar medium and sabouraud dextrose agar medium showing colony morphologies. **(B)** Concentrations of microbial colony forming units (CFU) in different PM samples during various haze-level days, Values shown as mean ± standard error (SEM).

The numbers of colonies were calculated in each agar plate and the concentration of the bacteria and fungi in the air were estimated. The most colonies were observed in TSP samples and the fewest in PM2.5 samples (Figure 1A), which indicated that the bigger PMs contained more culturable microbes. Otherwise, the numbers of bacterial colonies are more than the fungal colonies in all types of PM samples (Figure 1A). To explore the effect of haze pollutants on the concentration of culturable bacteria and fungi, we compared the numbers of CFU on various levels of AQI. The results showed that there were different patterns between bacteria and fungi (Figure 1B). The numbers of bacterial colonies descended at first and then increased with the haze-level increasing, which indicated that there were more culturable bacteria during non-haze days and heavy-haze days, and fewest during light-haze days. However, the number of fungal colonies showed a trend of decline, which suggested that fungi were most influenced by heavy-haze (Figure 1B).

### Bacterial and Fungal Isolates Differences in Various Media, PM Fractions, and Haze-Levels Samples

**FIGURE 2:**
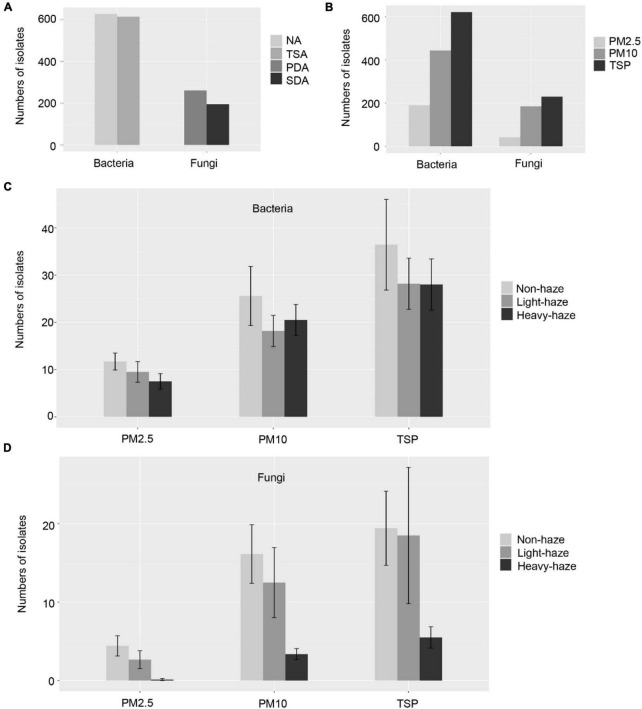
Numbers of microbial isolates in various media **(A)**, PM samples **(B)**, and haze-level samples **(C,D)**. Values are shown as mean ± standard error (SEM) in panels **(C,D)**.

Using four different media, we collected 1,258 bacterial and 456 fungal isolates with 637 bacterial isolates from NA medium, 621 bacterial isolates from TSA medium, 261 fungal isolates from PDA medium, and 195 fungal isolates from SDA medium (Figure 2A). Colonies were picked based on phenotypic diversity (representative plates shown in Figure 1A) with 194 bacterial isolates from PM2.5 samples, 443 bacterial isolates from PM10 samples, and 621 bacterial isolates from TSP samples, and with 41 fungal isolates from PM2.5 samples, 185 fungal isolates from PM10 samples, and 230 fungal isolates from TSP samples, which indicated the numbers of isolates increased with the increasing of the PM size and were consistent with the numbers of CFU in PM2.5, PM10, and TSP samples (Figure 2B). Furthermore, we compared the numbers of isolates among non-, light-, and heavy-haze days. With increase of the haze, the microbial isolates were decreased in all fractions of PM samples (Figures 2C,D). Especially, fungal isolates have sharp declines during heavy-haze days (Figure 2D).

### Diverse Bacterial and Fungal Representatives From Microbiome Associated With Particulate Matter

**FIGURE 3:**
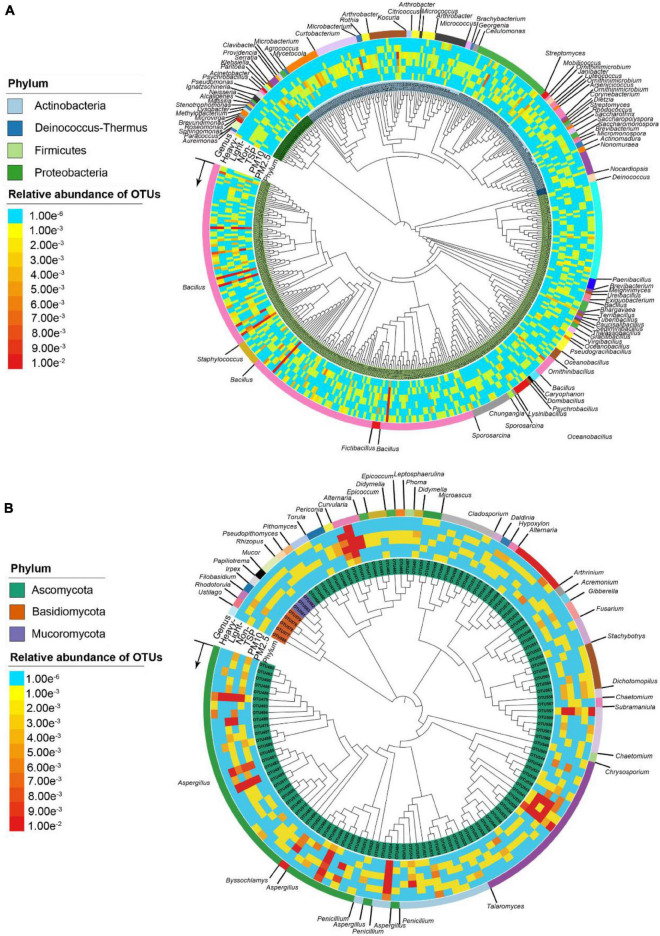
Phylogenetic tree of microbial isolates based on bacterial 16S rRNA sequences **(A)** and fungal ITS sequences **(B)** using neighbor-joining methods.

There were 1,258 bacterial isolates clustered into 455 OTUs (100% similarity). The culturable bacterial community associated with PM was dominated by the Firmicutes (930 isolates, 73.9%), followed by the Actinobacteria (283 isolates, 22.5%), Proteobacteria (41 isolates, 3.3%), and Deinococcus-Thermus (4 isolates, 0.32%) (Supplementary Table 4). There were 77 observed genera in the PM samples. *Bacillus* (750 isolates, 59.6%) was the most abundant genus, followed by *Streptomyces* (76 isolates, 6.0%), *Paenibacillus* (57 isolates, 4.5%), *Microbacterium* (35 isolates, 2.8%), *Curtobacterium* (34 isolates, 2.7%), *Kocuria* (34 isolates, 2.7%), and *Sporosarcina* (24 isolates, 2.0%) (Supplementary Table 5 and Figure 3A).

There were 475 fungal isolates clustered into 130 OTUs (100% similarity). The culturable fungal community associated with PM was dominated by the Ascomycota (466 isolates, 98.1%), followed by the Basidiomycota (6 isolates, 1.2%), and Mucoromycota (3 isolates, 0.6%) (Supplementary Table 6). There were 35 observed genera in PM samples. *Aspergillus* (126 isolates, 26.5%) was the most abundant genus, followed by *Penicillium* (105 isolates, 22.1%), *Talaromyces* (95 isolates, 20.0%), *Alternaria* (59 isolates, 12.4%), *Chaetomium* (22 isolates, 4.6%), *Dichotomopilus* (8 isolates, 1.7%), and *Arthrinium* (8 isolates, 1.7%) (Supplementary Table 7 and Figure 3B).

### Culturable Bacterial and Fungal Composition Among Various Particulate Matter Fractions and Among Non-, Light-, and Heavy-Haze Days

**FIGURE 4:**
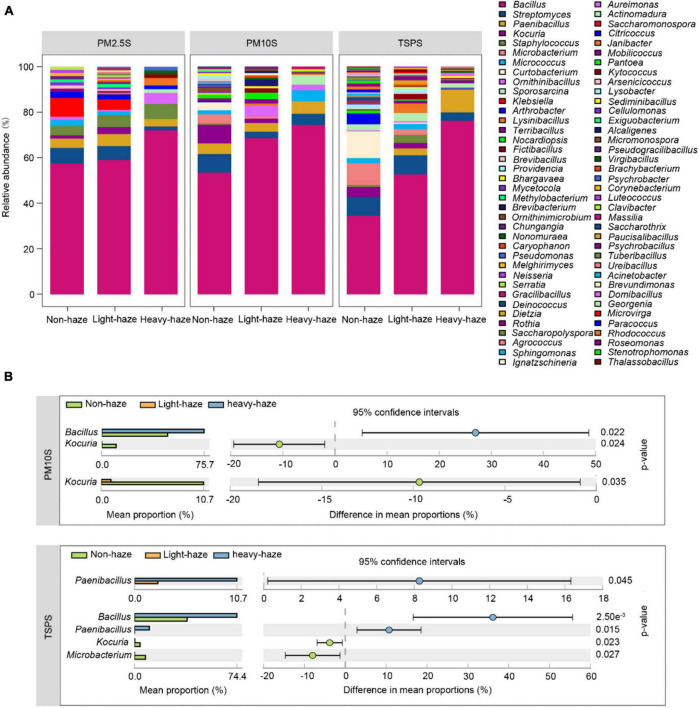
Bacterial diversity and composition among various PMs during non-, light-, and heavy-haze days. **(A)** Bar plot indicating relative abundances of bacterial genera among PMs. **(B)** STAMP analysis indicated genera that were significantly different among various haze-level samples.

Bar plots showed that the most abundant genus *Bacillus* was enriched during haze days with a haze-level dependent manner (Figure 4A). Surprisingly, *Staphylococcus* and *Klebsiella*, which include opportunistic pathogenic strains, were mainly isolated from PM2.5 samples, which increased the risk of infection in the upper respiratory tract (Figure 4A). Furthermore, *Staphylococcus* was enriched during haze days with a haze-level dependent manner in PM2.5 samples. Conversely, *Klebsiella* was enriched during non-haze days with a haze-level dependent manner in PM2.5 samples. We further identified the genera with significant differences among non-, light-, and heavy-haze samples using STAMP software (Figure 4B). No genera with significant differences were observed in PM2.5 samples. Compared to non-haze days, *Bacillus* was significantly enriched during heavy-haze days while *Kocuria* was significantly decreased during light- and heavy-haze days in PM10 samples (Figure 4B). In TSP samples, *Paenibacillus* was significantly enriched during heavy-haze days compared to light-haze days; *Bacillus* and *Paenibacillus* were significantly enriched during heavy-haze days while *Kocuria* and *Microbacterium* were significantly enriched during non-haze days (Figure 4B). CCA (Supplementary Figure 2A) and permutation tests (Supplementary Table 8) were performed to examine the relationships between bacterial community composition and environmental parameters. The concentration of SO_2_ (*r*^2^ = 0.2482, *p* ≤ 0.001), NO_2_ (*r*^2^ = 0.1447, *p* ≤ 0.05), CO (*r*^2^ = 0.1322, *p* ≤ 0.05), and the temperature (*r*^2^ = 0.1966, *p* ≤ 0.01) were significantly correlated with the bacterial community composition (Supplementary Figure 2A).

**FIGURE 5:**
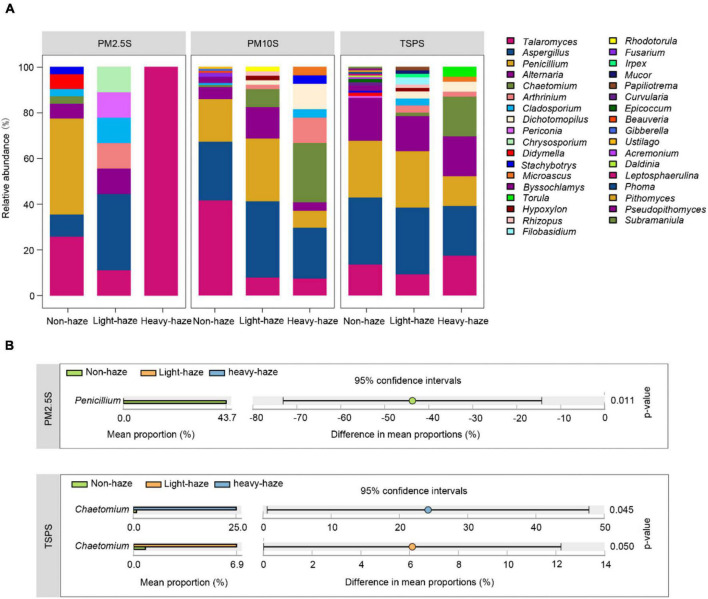
Fungal diversity and composition among various PMs during non-, light-, and heavy-haze days. **(A)** Bar plot indicating relative abundances of fungal genera among PMs. **(B)** STAMP analysis indicated genera that were significantly different among various haze-level samples.

The most abundant genera, *Talaromyces* and *Aspergillus*, have different patterns in various PM samples, which cannot be influenced by haze. For example, we only isolated *Talaromyces* in PM2.5 samples during heavy-haze days while it was enriched in PM10 samples during haze days (Figure 5A). *Penicillium* was enriched during non-haze days in a haze-level dependent manner. Especially in PM2.5 samples, *Penicillium* was only isolated during non-haze days, which indicated that it was easily inhibited by haze pollutants (Figures 5A,B). In addition, *Chaetomium* was significantly enriched during light- and heavy-haze days compared to non-haze days in TSP samples, which was similar to that in PM10 samples (Figures 5A,B). CCA (Supplementary Figure 2B) and permutation tests (Supplementary Table 9) indicated that the concentration of SO_2_ (*r*^2^ = 0.3973, *p* ≤ 0.001), CO (*r*^2^ = 0.5506, *p* ≤ 0.001), NO_2_ (*r*^2^ = 0.2679, *p* ≤ 0.01), and the temperature (*r*^2^ = 0.3410, *p* ≤ 0.01) were significantly correlated with the fungal composition (Supplementary Figure 2B).

### Antimicrobial Activities of Bacterial Representatives

To investigate the antimicrobial activity of microbial representatives in PM samples, we screened the microbial representatives of bacterial and fungal isolates. A total of 40 bacterial representatives had antimicrobial activity against *E. coli*, *E. faecalis*, *K. pneumoniae*, and *C. albicans*, while one fungal representative had antimicrobial activity.

**FIGURE 6:**
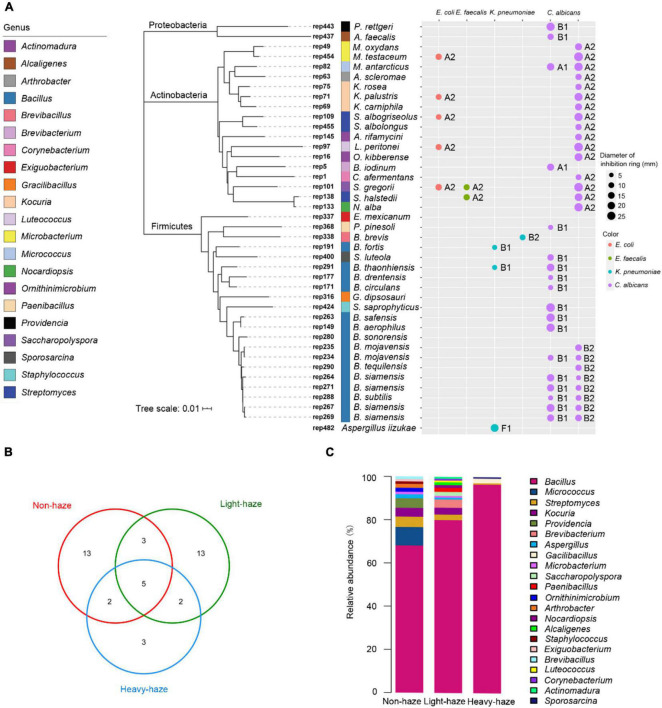
Antimicrobial activity of microbial representatives. **(A)** Phylogenetic tree of microbial isolates based on bacterial 16S rRNA sequences using neighbor-joining methods and activity patterns of microbial representatives with antimicrobial activity. **(B)** Venn diagram indicating unique representatives during various haze-level days. **(C)** Bar plot indicating relative abundances of genera with antimicrobial activity.

The microbial representatives with antimicrobial activity were dominated by the Firmicutes (21 representatives), followed by the Actinobacteria (17 representatives), and Proteobacteria (2 representatives) (Figure 6A). There were 22 genera with observed antimicrobial activity. *Bacillus* (15 representatives) was the most abundant genus, followed by *Kocuria* (3 representatives), *Microbacterium* (2 representatives), and *Streptomyces* (2 representatives) (Figure 6A). Anti-*E. coli* and anti-*E. faecalis* activities were only detected from fermentation broths of Actinobacteria, such as rep454 (closest to *Micrococcus testaceum*), rep71 (closest to *Kocuria palustris*), rep109 (closest to *Streptomyces albogriseolus*), rep97 (closest to *Luteococcus peritonei*), and rep101 (closest to *Saccharopolyspora gregorii*) with anti-*E. coli* activity, rep101 (closest to *Saccharopolyspora gregorii*), and rep138 (closest to *Streptomyces halstedii*) with anti-*E. faecalis* activity (Figure 6A). Fermentation broths of rep191, rep291, and rep338 (belong to *Bacillus*) and rep482 (closest to *Aspergillus iizukae*) have the activity to inhibit *K. pneumoniae* (Figure 6A). There were 35 representatives belonging to 19 genera that can inhibit the growth of *C. albicans*. Especially, both fermentation broths of rep82 (closest to *Micrococcus antarcticus*), rep234 (closest to *Bacillus mojavensis*), rep264, rep271, rep267, and rep269 (closest to *Bacillus siamensis*), and rep288 (closest to *Bacillus subtilis*) showed antimicrobial activity against *C. albicans* (Figure 6A).

The venn diagram indicates the unique representative with antimicrobial activity in various haze-level days. During non-haze days, 13 representatives (2 representatives were classified to *Bacillus*, 1 representative was classified to *Brevibacillus*) with antimicrobial activities were observed in PM samples (Figure 6B and Table 2). During light-haze days, 13 representatives (3 representatives were classified to *Bacillus*, 1 representative was classified to *Paenibacillus*) with antimicrobial activities were observed in PM samples (Figure 6B and Table 2). The fewest unique representatives with antimicrobial activities were observed during heavy-haze days, only 3 representatives (1 representative were classified to *Bacillus*, 1 representative was classified to *Gracilibacillus*) with antimicrobial activities were observed in PM samples, which suggested that the most abundant representatives with antimicrobial activities during heavy-haze days were classified to *Bacillus* while only a few ones during non- and light-haze days (Figure 6B and Table 2). Additionally, the fewest genera with antimicrobial activities were observed during heavy-haze days, for example, only *Bacillus*, *Streptomyces*, *Gacilibacillus*, and *Sporosarcina* were observed in PM samples (Figure 6C). To further investigate whether microbial community composition with antimicrobial activities is influenced by environmental parameters, CCA (Supplementary Figure 2C) and permutation test (Supplementary Table 10) were conducted. The results indicated that only the temperature (*r*^2^ = 0.2524, *p* ≤ 0.05) was significantly correlated with the microbial community composition with antimicrobial activities (Supplementary Figure 2C).

**TABLE 2:** The unique representatives with antimicrobial activity during various haze-level days.

Non-haze	Light-haze	Heavy-haze
rep16 (*Ornithinimicrobium*)	rep1 (*Corynebacterium*)	rep290 (*Bacillus*)
rep63 (*Arthrobacter*)	rep5 (*Brevibacterium*)	rep316 (*Gracilibacillus*)
rep71 (*Kocuria*)	rep49 (*Microbacterium*)	rep400 (*Sporosarcina*)
rep82 (*Micrococcus*)	rep69 (*Kocuria*)	
rep109 (*Streptomyces*)	rep97 (*Luteococcus*)	
rep191 (*Bacillus*)	rep101 (*Saccharopolyspora*)	
rep235 (*Bacillus*)	rep133 (*Nocardiopsis*)	
rep337 (*Exiguobacterium*)	rep145 (*Actinomadura*)	
rep338 (*Brevibacillus*)	rep177 (*Bacillus*)	
rep424 (*Staphylococcus*)	rep264 (*Bacillus*)	
rep443 (*Providencia*)	rep269 (*Bacillus*)	
rep454 (*Microbacterium*)	rep368 (*Paenibacillus*)	
rep455 (*Streptomyces*)	rep437 (*Alcaligenes*)	

## Discussion

Haze has been a major issue in north China, posing a substantial threat to public health. Recent studies revealed the diversity and composition of airborne microbial community during haze and non-haze days using culture-independent methods (Woo et al., 2013; Cao et al., 2014; Wei et al., 2016; Yan et al., 2016, 2018; Zhong et al., 2019; Yu et al., 2020). However, the airborne microbial community described with culture-independent methods cannot reflect the true function of microorganisms on human health and the environment. It is critical to investigate the culturable microorganisms in the PM, which enlarge our knowledge on the effects of haze on human health through airborne microorganisms. In this study, we isolated the airborne bacteria and fungi from three types of PM fractions, analyzed the concentration of culturable microorganisms during haze and non-haze days, and further revealed the anti-pathogen activities, which suggested the interactions between culturable microorganisms and common pathogens and help to find potential antimicrobial drugs in the airborne environment.

A lower concentration of fungi than bacteria was observed in the current study, which could be attributable to the samples we collected in autumn and winter. Previous studies reported that the fungal spore concentrations increased during summer and decreased sharply during autumn and winter (Haas et al., 2013; Gao et al., 2015). In our study, the concentrations of culturable bacteria and fungi showed different patterns in various haze levels. The lowest concentrations of airborne bacteria were detected on level 2 or level 3 days, while the highest ones were on level 1 or level 6. Yang et al. (2021) suggested that the mean bioaerosol concentrations were slightly higher during non-haze days than haze days, but there were no significant differences. Using an epifluorescence microscope after staining with the LIVE/DEAD^®^ BacLight™ Bacterial Viability Kit, Gong et al. (2019) reported that the bacterial viability decreased when air pollution occurred and increased again when pollution became severe, which is consistent with our study. According to Gong et al. (2019), during heavy-haze days, the concentrations of total airborne bacteria (including non-culturable and culturable bacteria) were 2–3 times greater than that during light- or non-haze days, explaining why the concentration of culturable bacteria increased. Thus, the growth of bacteria can be inhibited by haze, which accounts for the decrease of bacterial concentration during light-haze days. In addition, the lowest concentration of fungi was observed during heavy-haze days (level 5 and level 6). This is in line with previous findings (Gao et al., 2015), which suggested that haze might influence fungus spore germination.

The bacterial strains we isolated were far more than fungal strains, which confirmed that the fungi were hard to revive and culture in autumn and winter samples. Especially in PM2.5 samples, we only isolated 41 fungal strains compared to 185 and 230 strains in PM10 and TSP samples respectively, which might be due to the fact that most fungal spores have an aerodynamic diameter of > 2 mm (Froehlich-Nowoisky et al., 2009; Raisi et al., 2010; Haas et al., 2013). More microbial strains were isolated from non-haze samples, indicating that haze has an impact on airborne microorganisms. Particularly, we isolated relatively few fungal strains during heavy-haze days, implying that haze decreased more fungal isolates than bacterial ones.

Due to nutrition deficiency, dryness, and high levels of ultraviolet radiation in the air environment, most airborne microorganisms are non-viable in the form of dead cells, cell debris, or DNA fragments, resulting in a bias in the microbial community composition. It is a better strategy to estimate the composition of the airborne microbial community by combining the culture-dependent and culture-independent methods. The culturable bacterial community associated with PM was dominated by the Firmicutes (73.9%), Actinobacteria (22.5%), Proteobacteria (3.3%), and Deinococcus-Thermus (0.32%). With culture-independent methods, our group found that the bacterial community associated with PM was dominated by the Proteobacteria (38.5%), Firmicutes (26.8%), Actinobacteria (17.2%), Bacteroidetes (7.7%), and Deinococcus-Thermus (5.2%) (Yan et al., 2018), which suggested that Firmicutes were enriched by a culture-dependent method. Firmicutes is a phylum that includes all Gram-positive bacteria with a hard cell wall, making them resistant to haze and allowing them to thrive in the air (Garrity et al., 2009). Furthermore, the most abundant genus *Bacillus* was enriched during haze days in a haze-level dependent manner. *Bacillus* species have been detected in atmospheric dust (Verdona et al., 2013) and soil (Haas and Défago, 2005) producing oval endospores to adapt to the harsh environmental conditions and remain in a dormant state for a long period. In both PM10 and TSP samples, *Kocuria* was significantly enriched during non-haze days. In our previous study, *Kocuria* was identified as a key genus in the airborne PM samples, but no significant differences were observed during haze and non-haze days with culture-independent methods, which indicated that the survival of the genus can be influenced by haze pollutants (Yan et al., 2018). As microbial carriers, PM provided more surfaces for more airborne pathogenic bacteria to adhere to Li et al. (2015, 2017) and Xie et al. (2017). *Staphylococcus* and *Klebsiella*, including common pathogenic strains, were mainly isolated from PM2.5 samples, which increased the risk of infection in the upper respiratory tract. Especially, *Staphylococcus* was increased during haze days, reminding us to pay close attention to the infection by *Staphylococcus* during haze days. *Klebsiella*, which are recognized as human pathogens of the respiratory tract (Podschun and Ullmann, 1998), were found to be enriched in PM2.5 samples during non-haze days, implying that infection rates of *Klebsiella* were increased during non-haze days.

The composition of the culturable fungal community was altered in three types of PM fractions during haze days. *Talaromyces* was the most abundant genus in the present study, which is also different from the result with culture-independent methods, whereas *Cladosporium*, *Alternaria*, *Fusarium*, and *Penicillium* dominated the fungal community in our earlier study (Yan et al., 2016). *Talaromyces*, which is abundant in PM2.5 samples during haze days, contains species that are medically important and raise infection rates in the respiratory tract during hazy days (Yilmaz et al., 2014). For example, *T. marneffei* can cause a fatal mycosis in especially immunocompromised individuals from East Asian countries such as China, Taiwan, and Vietnam (Deng et al., 1988; Luh, 1998; Hien et al., 2001). Additionally, *Penicillium* was enriched during non-hazy days, which is similar to the results of a previous study using the culture-dependent method (Li et al., 2015). Haas et al. (2013) discovered that spore concentrations of *Penicillium*, rise with increased PM during non-haze days (Gao et al., 2015). Our previous study with the culture-independent method has indicated that the relative abundance of *Penicillium* increased during heavy-haze days (Yan et al., 2016). These findings suggested that haze pollutants might influence the germination or release of fungal spores. *Chaetomium* was enriched during haze days in the present study. Previous studies also reported *Chaetomium* stayed in the air (Shelton et al., 2002; David et al., 2013), and it could affect air quality and damage to human health (Nielsen et al., 1998, 1999; Guo et al., 2019), indicating that haze enhanced the disease risk caused by *Chaetomium*.

Although soils are considered excellent sources for the isolation of microorganisms with diverse potential, the current focus is on exploring previously ignored ecosystems (Lee and Hwang, 2002; Prabavathy et al., 2006; Shah et al., 2017). A scarce number of studies involving microorganisms collected from the air in antimicrobial activity screening (Sobral et al., 2017) and the diverse microbial community associated with PM was observed in the current and previous studies (Yan et al., 2016, 2018; Romano et al., 2020; Yu et al., 2020). The wide-ranging and fluctuating temperatures, high levels of solar irradiation, strong oxidizing, and dryness in the atmosphere may select for novel microorganisms with unique metabolic properties (Polymenakou, 2012). The percentage of representatives with antimicrobial activities decreased during non-haze days and the genera with antimicrobial activities were distributed in various haze-level samples, which indicated that haze pollutants may one of the factors to select for the strains with antimicrobial activities. Though the strains with antimicrobial activities can inhibit the growth of pathogens, the potential drug-resistant bacteria or fungi can be selected in the air, which may promote the dissemination of antibiotic resistance genes (ARGs) and influence public health due to the persistent haze. Zhu et al. (2021) also suggested that the dissemination of ARGs in fresh snow could be exacerbated by air pollution, severely increasing the health risks of both air pollution and ARGs. *Bacillus* was the most abundant airborne genus with microbial activity in this study. El-Banna (2005) isolated airborne microorganisms and five strains belonging to *Bacillus* were found to be antagonistic to bacteria or fungi. It is necessary to search for new antibiotics from *Bacillus*, which is one of the fruitful sources of antibiotics (Emmert et al., 2004; El-Banna, 2005). Actinobacteria, which were the abundant phylum with microbial activity from PM samples in the present study, have been the source of countless drugs and intensively screened as an important source of therapeutically important molecules for over half a century. For example, *Streptomyces* strains are the richest source of natural products, especially clinically useful antibiotics, antimetabolites, and antitumor agents (Bérdy, 2005; Newman and Cragg, 2007; Olano et al., 2018). Filamentous actinobacteria account for about 45% of all microbial bioactive secondary metabolites with about 80% of these 7,600 compounds being produced by *Streptomyces* (Bérdy, 2005; Goodfellow et al., 2009). Additionally, only one fungal strain belonging to *Aspergillus* showed antimicrobial activity, which suggested that the airborne fungi had a weak activity and the genus *Aspergillus* was the most prolific among fungi (Giordano, 2020).

## Conclusion

Our study provided an integrated characterization of the isolation and antimicrobial activities of bacterial and fungal representatives associated with PM during haze and non-haze days using a culture-dependent method. The numbers and composition of culturable bacteria and fungi were influenced by haze pollutants, which further enhances the understanding of the airborne microorganism during haze and non-haze days. We collected 1,258 bacterial isolates and 475 fungal isolates and increased the number of microbial resources for screening active strains. Additionally, 40 bacterial representatives and 1 fungal representative had antimicrobial activities against common pathogens, paving the way for the discovery of novel antibiotics. This study could help researchers better comprehend the airborne microbial resource during haze and non-haze days. Through further genomic characterization and analysis, separation of natural active products, and synthetic biology endeavors, we believe that the collection of airborne strains is a resource that will provide a basis to increase our understanding of the relationship between microbes associated with PM and human health.

## Data Availability Statement

The data that support the findings of this study are available in Supplementary Table 2. The raw reads were deposited into the NCBI GeneBank database under accession number SUB10504922 (OK482088–OK482542) and SUB10505231 (OK490148–OK490277).

## Author Contributions

L-YY designed the study and revised the manuscript. DY, JS, L-LZ, HW, and X-MF performed sampling. DY performed the laboratory work and wrote the manuscript. TZ and J-LB performed part of the laboratory work and revised the manuscript. DY, Y-QZ, and H-YL analyzed the data. All authors read and approved the final manuscript.

## Conflict of Interest

The authors declare that the research was conducted in the absence of any commercial or financial relationships that could be construed as a potential conflict of interest.

## Publisher’s Note

All claims expressed in this article are solely those of the authors and do not necessarily represent those of their affiliated organizations, or those of the publisher, the editors and the reviewers. Any product that may be evaluated in this article, or claim that may be made by its manufacturer, is not guaranteed or endorsed by the publisher.

## Footnotes

1
http://zx.bjmemc.com.cn/

2
http://www.nmc.cn/

3
https://www.ezbiocloud.net/

4
https://itol.embl.de
