# Supplementary material for: Isolation, Characterization, and Antimicrobial Activity of Bacterial and Fungal Representatives Associated With Particulate Matter During Haze and Non-haze Days
Source: Front Microbiol. 2022 Jan 11;12:793037. doi: 10.3389/fmicb.2021.793037 (PMC8787346; doi:10.3389/fmicb.2021.793037)
Supplement: Supplementary file 2 [file Data_Sheet_2.docx]

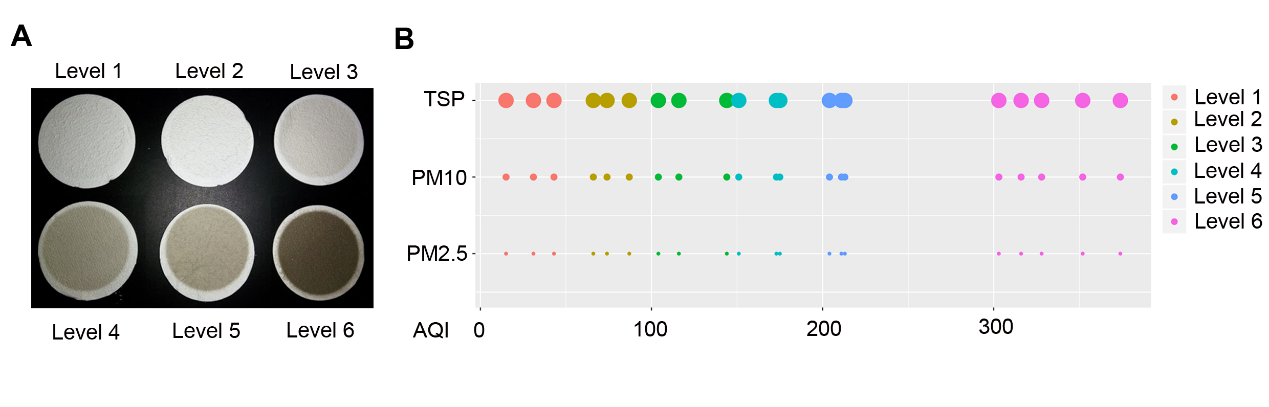


**Figure S1** (**A**) Quartz filters collected in PM2.5 samples during various haze-level days. (**B**) The distribution of different PM samples collected during various haze-level days.


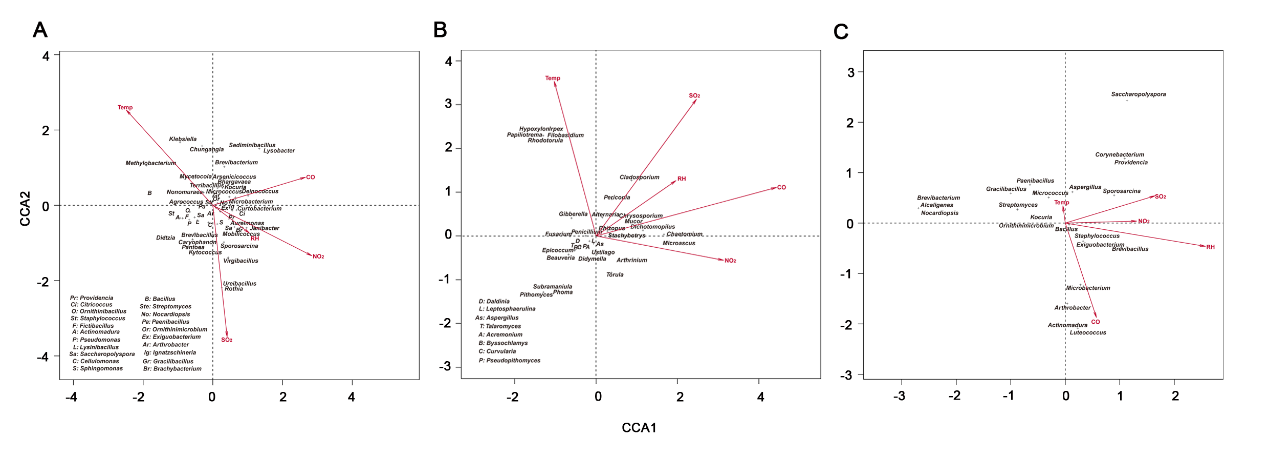


**Figure S2** (**A**) CCA between bacterial composition and environmental parameters. (**B**) CCA between fungal composition and environmental parameters. (**C**) CCA between microbial composition with antimicrobial activities and environmental parameters.
